# Hepatic microbiome in healthy lean and obese humans

**DOI:** 10.1016/j.jhepr.2021.100299

**Published:** 2021-04-27

**Authors:** Malte Palm Suppli, Jonatan Ising Bagger, Benjamin Lelouvier, Amandine Broha, Mia Demant, Merete Juhl Kønig, Charlotte Strandberg, Asger Lund, Tina Vilsbøll, Filip Krag Knop

**Affiliations:** 1 Center for Clinical Metabolic Research, Gentofte Hospital, University of Copenhagen, Hellerup, Denmark; 2 Vaiomer, Labège, France; 3 Department of Radiology, Gentofte Hospital, University of Copenhagen, Hellerup, Denmark; 4 Department of Clinical Medicine, Faculty of Health and Medical Sciences, University of Copenhagen, Copenhagen, Denmark; 5 Steno Diabetes Center Copenhagen, Gentofte, Denmark; 6 Novo Nordisk Foundation Center for Basic Metabolic Research, Faculty of Health and Medical Sciences, University of Copenhagen, Copenhagen, Denmark

**Keywords:** Non-alcoholic fatty liver disease, Obesity, Metabolic syndrome, Microbiome, FLI, fatty liver index, HbA1c, glycated haemoglobin, LEfSe, linear discriminant analysis effect size, NAFLD, non-alcoholic fatty liver disease, NASH, non-alcoholic steatohepatitis, OTU, operational taxonomic unit, qPCR, quantitative polymerase chain reaction, rDNA, ribosomal DNA

## Abstract

**Background & Aims:**

Dysbiosis of the gut microbiota in response to an energy-rich Western diet and the potential leak of bacteria and/or bacterial products from the intestine to the liver is perceived as a potential risk factor for the development of non-alcoholic fatty liver disease (NAFLD). We investigated the microbiome in liver biopsies from healthy lean and obese individuals and compared it with their blood microbiome.

**Methods:**

We examined liver biopsies from 15 healthy lean and 14 obese individuals (BMI of 18.5–25 and 30–40 kg/m^2^, respectively). Bacterial 16S ribosomal DNA (rDNA) was analysed by quantitative polymerase chain reaction (qPCR) and 16S metagenomic sequencing targeting the hypervariable V3–V4 region. Metagenomic analysis was performed using the linear discriminant analysis effect size (LEfSe) algorithm. Data are medians with IQRs in brackets.

**Results:**

Histology revealed hepatic steatosis in 13 obese individuals and in 2 lean individuals. A robust signal from qPCR revealed significantly higher amounts of bacterial rDNA copies in liver samples from obese individuals compared with those from lean individuals (148 [118–167] *vs*. 77 [62–122] 16S copies/ng DNA, *p* <0.001). Liver biopsies from the obese group were characterised by lower alpha diversity at the phylum level (Shannon index 0.60 [0.55–0.76] *vs.* 0.73 [0.62–0.90], *p* = 0.025), and metagenomic profiling revealed a significantly higher proportion of Proteobacteria in this group (81.0% [73.0–82.4%] *vs*. 74.3% [68.4–78.4%], *p* = 0.014).

**Conclusions:**

We provide evidence for the presence of bacterial rDNA in the healthy human liver. Based on differences in the hepatic microbiome between obese individuals and healthy lean individuals, we suggest that changes in the liver microbiome could constitute an additional risk factor for the development of NAFLD.

**Lay summary:**

Non-alcoholic fatty liver disease (NAFLD) has become the most common liver disease globally, and new evidence suggests that obesity is associated with a disturbed gut bacterial composition, which may influence the development of NAFLD. We examined the composition of bacterial DNA in liver biopsies from healthy lean and obese individuals and found a different composition of bacterial DNA in liver biopsies from the obese group. We propose that the increased bacterial DNA load in the livers of obese individuals could constitute an early risk factor for the progression of NAFLD.

**Clinical trial number:**

NCT02337660

## Highlights

• Liver tissue from healthy lean and obese individuals contains bacterial rDNA.
• Significantly higher amounts of bacterial rDNA and lower alpha diversity were found in liver samples from obese individuals.
• Proteobacteria-enriched metataxonomic signature was found in liver samples from obese individuals.
• There is strong correlation between the amount of bacterial rDNA in liver samples and the fatty liver index.
• Differences in the liver microbiome between lean and obese individuals are not reflected in the blood microbiome.

## Graphical abstract

**Figure:**
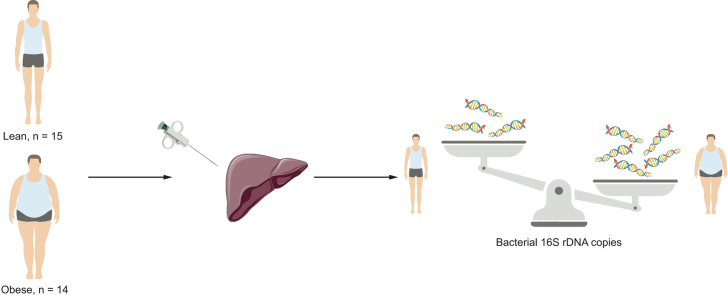

## Introduction

Non-alcoholic fatty liver disease (NAFLD) has become the most common liver disease with an estimated global prevalence of 25%.^1^ NAFLD constitutes a spectrum of conditions ranging from simple, benign, and reversible steatosis over non-fibrotic non-alcoholic steatohepatitis (NASH) to NASH with fibrosis that ultimately can progress to cirrhosis and end-stage liver disease. NAFLD is now considered the second most common cause of liver transplantation in the United States, only surpassed by hepatitis C,^2^ and, additionally, NAFLD greatly increases the risk of hepatocellular carcinoma.^3^^,^^4^ The increase in NAFLD parallels the global rise in obesity, and the metabolic syndrome is the strongest risk factor for the development of the disease.^5^^,^^6^ The pathophysiology of NAFLD is closely linked to chronically high caloric intake promoting continuous deposition of nutrients as fat in hepatocytes. Obesity-related insulin resistance and the ensuing hyperinsulinaemia exacerbates the deposition of lipids in the liver and, thus, contributes to the development of NAFLD.^7^ In this chronic ‘fed state’ without turnover of hepatic fat content, steatosis-induced hypoxia, cell death, infiltration of macrophages, and expression of chemokines and proinflammatory cytokines are considered major determinants for the transition from simple steatosis to NASH with ensuing fibrogenesis.^5^ However, the exact contributing pathogenic drivers of this transition are not fully understood.

Dysbiosis of the gut microbiota in response to an energy-rich Western diet is considered a contributing factor in the development of obesity.^8^ Thus, the phenotype of obese mice was transmissible to germ-free mice through inoculation with gut microbiota.^9^ Furthermore, high-fat diet-associated dysbiosis of the gut entails increased permeability of the intestinal epithelium through disruption of tight junctions and the mucus layer,^10^^,^^11^ leading to increased translocation of bacteria and bacterial products to the portal venous system.^12^^,^^13^ The liver is uniquely positioned as all portal blood from the gastrointestinal tract must pass through the liver before entering the systemic circulation. An increasing body of evidence suggests that changes in the gut microbiota plays an important role in the pathogenesis of NAFLD, and, importantly, in the transition from simple steatosis to NASH.^7^^,^^14^^,^^15^ In the liver, bacteria and bacterial products are thought to contribute to inflammation and fibrogenesis by acting as ligands for Toll-like receptors on Kupffer cells and hepatic stellate cells.^12^^,^^13^^,^^16^ Gram-negative bacteria from the Proteobacteria phylum, in particular, contribute to this process owing to their membrane content of lipopolysaccharides, which are known activators of Toll-like receptors.^17^ A recent characterisation of the blood microbiome in healthy individuals revealed that it is dominated by Proteobacteria, contrasting the gut microbiome, which is mainly dominated by Firmicutes and Bacteroidetes.^18^^,^^19^ This difference implies that a filtration takes place during the translocation of bacteria and bacterial products from the intestine to the systemic circulation, and the liver constitutes an important point of interest in this setting.^20^^,^^21^ However, most evidence implicating the gut–liver axis in NAFLD pathogenesis is derived from animal studies.

In the present study, we used both quantitative and qualitative DNA-based methods to provide a unique characterisation of the microbiome in the livers of obese individuals and healthy lean controls and compare it with their blood microbiome. DNA from blood and liver samples was isolated and amplified in a strictly controlled environment using a stringent contamination-aware approach described and discussed previously.^18^^,^[22], [23], [24] We report an overabundance of bacterial ribosomal DNA (rDNA) in the livers of obese individuals with hepatic steatosis compared with that of lean individuals, primarily driven by Proteobacteria.

## Materials and methods

### Ethical approval

The study was approved by the Research Ethics Committee of the Capital Region of Denmark (Reg. No. H-6-2014-097), registered with Clinicaltrials.gov (Registration No. NCT02337660), and was conducted in accordance with the latest revision of the Declaration of Helsinki. A liver biopsy was performed on each participant. Subsequently the study participants took part in an experimental day. Data from the experimental day and from RNA sequencing of the liver biopsies have been reported previously.[25], [26], [27]

### Study participants

Study participants were recruited from the Greater Copenhagen area and studied at Gentofte Hospital, University of Copenhagen, Hellerup, Denmark. We included 30 healthy males between 25 and 80 years of age for a clinical study and performed the liver biopsy procedure as a part of this study.^25^ Participation in the clinical study was the only indication for performing the liver biopsy, and the participants were recruited for this purpose alone. None of the participants showed clinical signs of liver disease. The study (including the liver biopsy) was approved by the Danish Research Ethics Committees, and the participants gave their informed consent after receiving information about the clinical study and the liver biopsy both orally and written. Exclusion criteria included known liver disease, alanine aminotransferase and/or aspartate aminotransferase >2× the upper reference limits (*i.e.* alanine aminotransferase >140 U/L and/or aspartate aminotransferase >90 U/L), weekly alcohol consumption >14 U/week, pre-diabetes (glycated haemoglobin [HbA1c] ≥42 mmol/mol), diabetes or first-degree relatives with diabetes. One participant was excluded from the analysis (see the Statistical Analysis section). The study participants are characterised in Table 1.

**Table 1:** **Study participant characteristics for lean (BMI 18.5–25 kg/m**^**2**^**) and obese (BMI 30–40 kg/m**^**2**^**) healthy individuals**.

	Lean (n = 15)	Obese (n = 14)	*p* value
Age (years)	40 (25–68)	32 (25–58)	0.315
Sex (male)	15	14	–
BMI (kg/m^2^)	23.8 (20.7–25.0)	33.4 (30.9–39.8)	<0.0001
FPG (mmol/L)	5.5 (4.9–5.9)	5.6 (4.7–6.1)	0.292
Waist–hip ratio	0.86 (0.80–0.98)	1.0 (0.89–1.1)	<0.0001
HbA1c (mmol/mol)	30 (23–34)	33 (26–37)	0.344
C-peptide (pmol/L)	309 (198–697)	769 (287–1200)	<0.0001
ALT (U/L)	31 (21–55)	38 (18–70)	0.213
AST (U/L)	32 (21–53)	38 (25–83)	0.119
Triglycerides (mmol/L)	1.1 (0.5–2.3)	2.2 (0.8–8.1)	0.001
Total cholesterol (mmol/L)	4.5 (3.5–5.8)	5.2 (3.5–8.8)	0.0374
FLI	23.1 (4.1–65.1)	89.5 (60.2–99.2)	*<*0.0001

Data are presented as medians with ranges in brackets. The groups were compared using the Mann–Whitney *U* test. ALT, alanine aminotransferase; AST, aspartate aminotransferase; FLI, fatty liver index; FPG, fasting plasma glucose; HbA1c, glycated haemoglobin.

### Liver biopsies and buffy coat

For the evaluation of hepatic steatosis, fibrosis, and inflammation, an ultrasound-guided liver biopsy was performed on each participant by a specialised radiologist in the Department of Radiology, Gentofte Hospital, University of Copenhagen, Hellerup, Denmark. Biopsies were taken in the morning after an overnight 10-h fast, including coffee and tobacco. The biopsy was collected using a sterile BARD® MONOPTY® Disposable Core Biopsy Instrument (Bard Biopsy, Tempe, AZ, USA) with a gauge size of 14 g (2.0 cm) and a penetration depth of 22 mm. The insertion site was cleaned by double-wiping of the skin with ethanol according to surgical protocol, and the abdomen of the participants was covered with a sterile drape before the procedure. The biopsy was immediately divided and distributed into formalin or snap-frozen in liquid nitrogen using sterile instruments. Tubes with formalin were kept on ice and stored at −20°C, and snap-frozen samples were stored at −80°C. None of the participants experienced complications in relation to the liver biopsy. Histological examination was performed by the same pathologist on formalin-fixed, paraffin-embedded liver sections at the Department of Pathology, Herlev Hospital, University of Copenhagen, Herlev, Denmark. Blood for the collection of buffy coat was collected in chilled tubes containing EDTA and subsequently centrifuged for 20 min at 1,200 *g* and 4°C. Buffy coat samples were stored at −20°C.

### 16S rDNA quantitation and metagenomic profiling

DNA from blood and liver biopsies was isolated and amplified in a strictly controlled environment at Vaiomer SAS (Labège, France) using a stringent contamination-aware approach as discussed previously.^18^^,^[22], [23], [24] Real-time quantitative polymerase chain reaction (qPCR) amplification was performed using 16S universal primers targeting the hypervariable V3–V4 region of the bacterial 16S ribosomal gene as described previously.^18^^,^^19^^,^^22^ The qPCR step was performed on a ViiA 7® PCR system (Life Technologies, Carlsbad, CA, USA) using Sybr Green technology. The microbial populations based on rDNA present in liver samples and buffy coat were determined using next-generation sequencing of V3–V4 variable regions of the 16S rRNA bacterial gene as previously described.^22^

For each sample, a sequencing library was generated by addition of sequencing adapters. The joint pair length was set to encompass a 467 base pairs amplicon (using *Escherichia coli* 16S as a reference) with a 2×300 paired-end MiSeq kit V3 (Illumina, San Diego, CA, USA). The detection of the sequencing fragments was performed using the MiSeq Illumina® technology.

Targeted metagenomic sequences from microbiota were analysed using the bioinformatic pipeline from the FROGS guidelines.^28^ Briefly, the cleaning was done by removing amplicons without the 2 PCR primers (10% of mismatches were authorised), amplicons with at least 1 ambiguous nucleotide (‘N’), amplicons identified as chimera (with vsearch v1.9.5), and amplicons with a strong similarity (coverage and identity ≥80%) with the phiX (library used as a control for Illumina sequencing runs). Clustering was produced in 2 passes of the swarm algorithm v2.1.6. The first pass was a clustering with an aggregation distance equal to 1. The second pass was a clustering with an aggregation distance equal to 3. Taxonomic assignment of amplicons into operational taxonomic units (OTUs) was produced by Blast+ v2.2.30+ with the Silva 128 Parc databank. To assess if the richness of microbiota was adequately captured by metagenomic sequencing, a rarefaction analysis was performed. To ensure a low background signal from bacterial contamination of reagents and consumables, 2 types of negative controls consisting of molecular-grade water were added in an empty tube separately at the DNA extraction step and at the PCR steps and amplified and sequenced at the same time as the extracted DNA of the blood samples. Both the beta diversity analysis and the qPCR analysis show a clear separation between negative controls, and both blood samples and liver samples (Fig. S1A–C). These controls confirm that bacterial contamination was well contained in our pipeline and had a negligible impact on the taxonomic profiles of the samples of this study as we published before.^18^^,^[22], [23], [24] The sequencing quality was high. The number of raw pairs was around 50,000 per sample. Both reads were trimmed of 10 base pairs (2×290 base pairs used for joining). An average of 78.8% of reads per sample were successfully joined, and only 4.3% of the sequences were identified as chimera and excluded. On average, 35,000 reads per sample were clustered in OTUs, and 98.9% of them were successfully affiliated to a bacterial taxon. The number of sequences classified to OTUs were similar between samples—around 35,000 per sample (from around 50,000 raw pairs of reads, *i.e.* 100,000 total reads per sample) (Fig. S2).

### Statistical analysis

Statistical analyses were performed using the software GraphPad Prism (v8.0.0, GraphPad Software, San Diego, CA, USA). Unless otherwise stated, results are presented as medians with IQRs in brackets. Mann–Whitney *U* tests were used to compare the 2 groups. Regression analyses were carried out in the statistical software R (v. 3.5.2). The amount of bacterial 16S rDNA copies/ng DNA in the liver samples was used as the dependent variable, and anthropometric and biochemical data were used as independent variables. Initially, a simple linear regression was performed for all independent variables, *i.e.* BMI, age, waist–hip ratio, fasting plasma glucose, C-peptide, HbA1c, homoeostatic model assessment of insulin resistance, alkaline phosphatase, alanine aminotransferase, aspartate aminotransferase, bilirubin, triglycerides, cholesterol, and the fatty liver index (FLI) (a validated marker of NAFLD based on BMI, waist–hip ratio, gamma-glutamyltransferase, and triglycerides). Correlations among the independent variables were analysed to avoid multicollinearity. Values of *p* <0.05 were considered significant. One individual from the obese group was identified as an outlier and was excluded from the analyses. Because of the exploratory nature of the study with limited group sizes and, hence, limited statistical power, we did not correct for multiple testing in the metagenomic analyses. Naturally, this limits our ability to draw final conclusions as discussed below.

## Results

### Liver histology

Histopathological scoring of the liver biopsies was performed with a focus on the degree of steatosis, lobular inflammation, hepatocyte ballooning, and fibrosis. Benign steatosis was found in 2 lean participants (7% and 40% steatosis), whereas it was present in 13 of 14 obese individuals covering a spectrum from less than 5% to 40% steatosis. Thus, in the obese group, 7 participants met the definition of NAFLD with more than 5% steatosis. None of the participants had histological evidence of lobular inflammation, hepatocyte ballooning, or fibrosis.

### Bacterial 16S rDNA is more abundant in liver samples from obese individuals

**Fig. 1:**
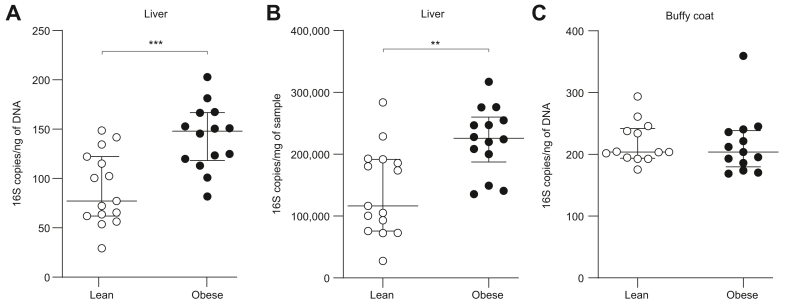
Bacterial 16S rDNA is more abundant in liver samples from obese individuals than in lean controls. Median ± IQR (whiskers plot) and individual values (dot plot) of 16S ribosomal DNA assessed by real-time PCR amplification per (A) ng of DNA and (B) mg of samples in liver tissue and (C) per ng of DNA in buffy coat in lean (open symbols) and obese individuals (filled symbols). ∗∗*p* <0.01, ∗∗∗*p* <0.001 (Mann–Whitney *U* test). rDNA, ribosomal DNA.

The quantitation of bacterial 16S rDNA revealed significantly higher amounts of 16S copies in liver samples from the obese group compared with those from lean individuals. The difference was found both relative to the total amount of DNA (148 [118–167] *vs*. 77 [62–122] 16S copies/ng DNA, *p* <0.001) and to the weight of the samples (225,990 [187,473–260,087] *vs*. 116,557 [75,785–191,955] 16S copies/mg sample, *p* <0.01) (Fig. 1A,B). Conversely, the amount of 16S copies in buffy coat (containing on average more than 93% of blood bacterial rDNA^18^) was similar between lean and obese individuals (204 [180–238] *vs*. 204 [194–242] 16S copies/ng DNA, *p* = 0.58) (Fig. 1C).

### Obese individuals have lower 16S rDNA alpha diversity in the liver at the phylum level

**Fig. 2:**
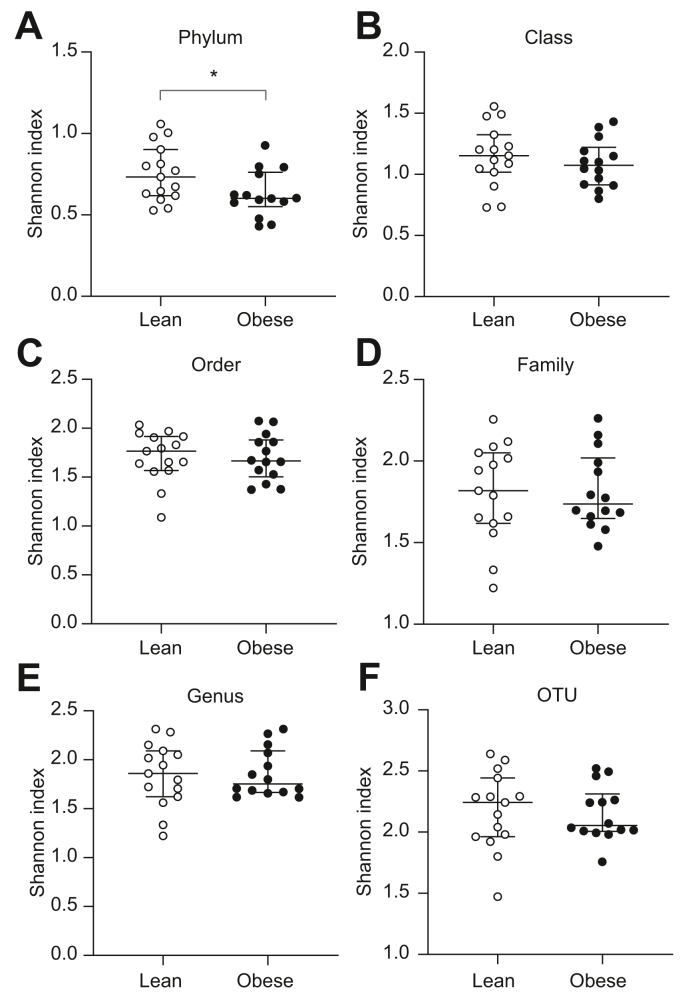
Lower bacterial alpha diversity at the phylum level in liver samples from obese individuals. Median ± IQR (whiskers plot) and individual values (dot plot) of bacterial alpha diversity (Shannon index) at the level of (A) phylum, (B) class, (C) order, (D) family, (E) genus, and (F) OTU assessed by 16S metagenomics sequencing in the livers of lean (open symbols) and obese individuals (filled symbols). ∗*p* <0.05 (Mann–Whitney *U* test). OTU, operational taxonomic unit.

**Fig. 3:**
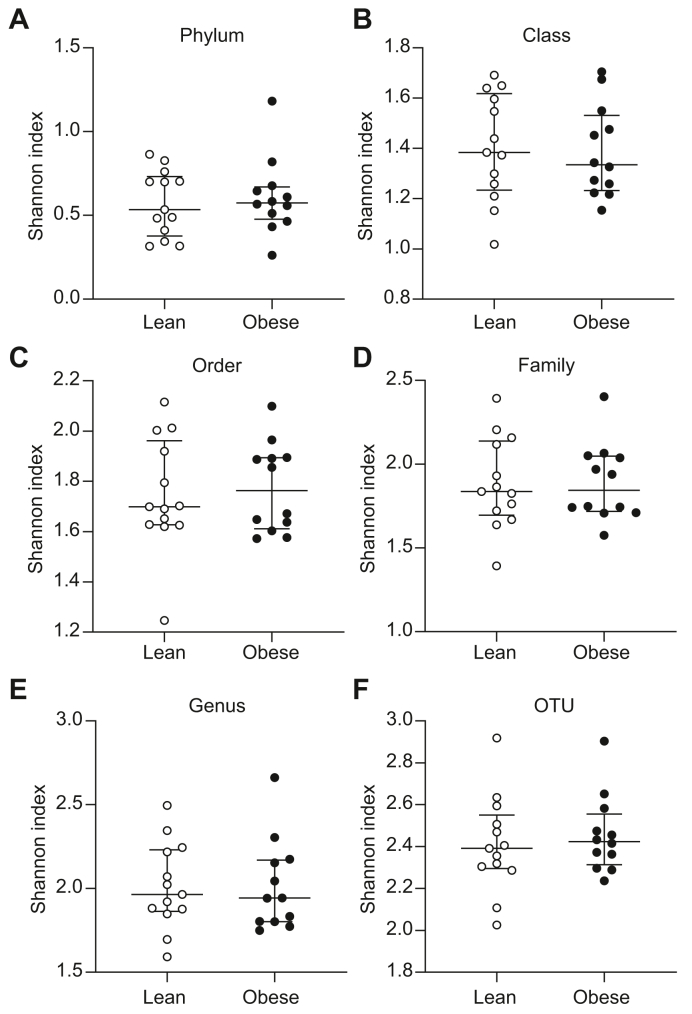
No difference in bacterial alpha diversity in buffy coat between lean and obese individuals. Median ± IQR (whiskers plot) and individual values (dot plot) of bacterial alpha diversity (Shannon index) at the level of (A) phylum, (B) class, (C) order, (D) family, (E) genus, and (F) OTU assessed by 16S metagenomics sequencing in buffy coat from lean (open symbols) and obese individuals (filled symbols). The groups were compared using the Mann–Whitney *U* test. OTU, operational taxonomic unit.

We then assessed whether the increased abundance of bacterial rDNA in obese individuals was associated with a change in the taxonomic diversity in the samples. The Shannon index, which is a measure of bacterial alpha diversity (*i.e.* taxa richness and evenness in a sample), was calculated after 16S-targeted metagenomic sequencing of the 16S rDNA. At the phylum level, bacterial alpha diversity was significantly lower in the obese group than in the lean group (Shannon index 0.60 [0.55–0.76] *vs.* 0.73 [0.62–0.90], *p* = 0.025) (Fig. 2A), whereas there were no differences in the alpha diversity between the groups at the level of class, order, family, genus, or OTUs (Fig. 2B–F). There were no differences in the bacterial alpha diversity in buffy coat between the groups at any of the taxonomic levels (Fig. 3A–F). Ordination figures based on Bray–Curtis distance analysis of the beta diversity are provided in Fig. S3. As expected, because of the high interindividual variability between participants, almost no shift between the biological groups could be observed in the ordination.

### Proteobacteria are more abundant in liver samples from obese individuals than in those from lean controls

**Fig. 4:**
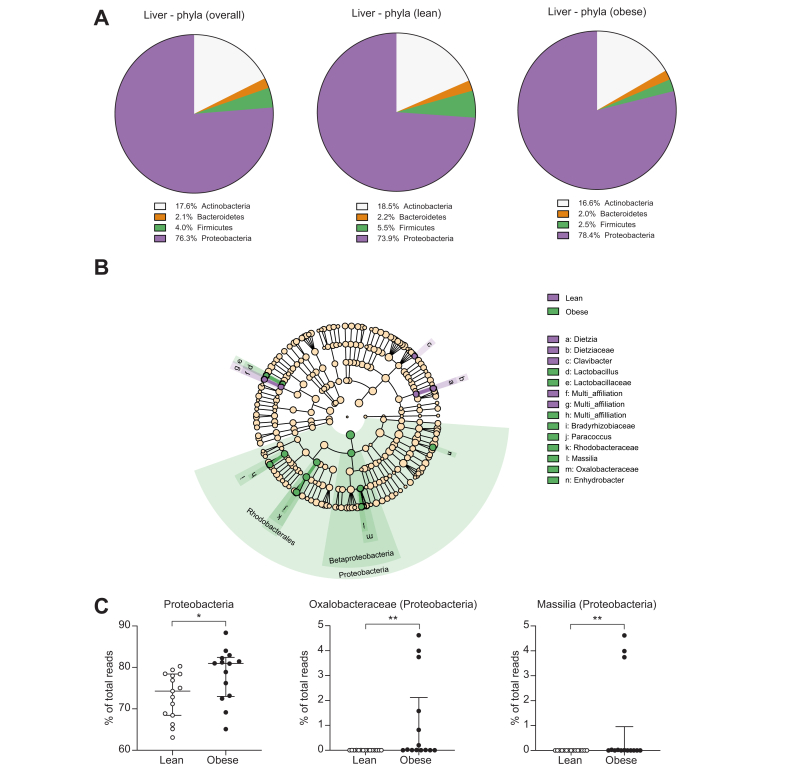
Proteobacteria are more abundant in liver samples from obese individuals than in those from lean individuals. Bacterial profiles assessed by 16S metagenomics sequencing of liver biopsies from lean and obese individuals. (A) Mean bacterial composition at the phylum level in the study groups. (B) LEfSe cladogram showing taxa more abundant in the lean group (red) and in the obese group (green); in the output of the bioinformatic pipeline, ‘unknown’ refers to sequences without information for the given taxonomic level in the 16S database, whereas ‘multi-affiliation’ refers to sequences found in the database, but with several possible taxa at the given taxonomic level. The multi-affiliated genus ‘h’ belongs to the Bradyrhizobiaceae family, and genus ‘f’ and family ‘g’ belong to the Lactobacillales order. (C) Median ± IQR (whiskers plot) and individual values (dot plot) of relevant bacterial taxa in lean (open symbols) and obese individuals (filled symbols). ∗*p* <0.05, ∗∗*p* <0.01 (Mann–Whitney *U* test). The significance levels for Oxalobacteraceae and *Massilia* should be taken with caution owing to zero-inflation of the data.

**Fig. 5:**
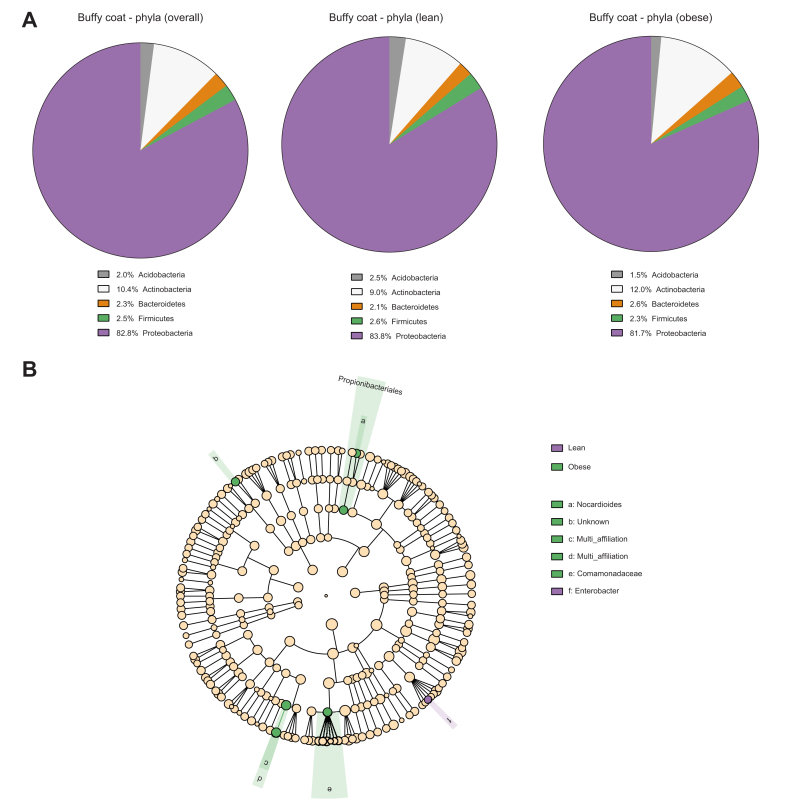
Similar bacterial metagenomic profiles in buffy coat between lean and obese individuals. Bacterial profiles assessed by 16S metagenomics sequencing in buffy coat from lean and obese individuals. (A) Mean bacterial composition at the phylum level in the study groups. (B) LEfSe Cladogram showing taxa more abundant in the lean group (red) and in the obese group (green); in the output of the bioinformatic pipeline, ‘unknown’ refers to sequences without information for the given taxonomic level in the 16S database, whereas ‘multi-affiliation’ refers to sequences found in the database, but with several possible taxa at the given taxonomic level. The unknown genus ‘b’ belongs to the Chitinophagaceae family, but it was not considered a pertinent taxon as the difference was driven by an outlier sample among zero-inflated data. The multi-affiliated genus ‘c’ and family ‘d’ belong to the Sphingomonadales order.

By performing taxonomic assignment of the bacterial 16S rDNA profiles, we found that the overall hepatic bacterial composition in the study population consisted mainly of Proteobacteria (76.3%) and Actinobacteria (17.6%) and, to a lesser extent, of Firmicutes (4.0%) and Bacteroidetes (2.1%) (Fig. 4A and Fig. S4). Interestingly, the proportion of Proteobacteria seemed higher in the obese group than in the lean group (mean proportion 78.4% *vs.* 73.9%), whereas the proportion of Firmicutes was lower in the obese individuals (mean proportion 2.5% *vs.* 5.5%) (Fig. 4A and Fig. S4). The metagenomic profiles of the liver samples were then further analysed using the linear discriminant analysis effect size (LEfSe) algorithm (Fig. 4B) and the Mann–Whitney *U* test (Fig. 4C). In the output of the bioinformatic pipeline, ‘unknown’ refers to sequences without information for the given taxonomic level in the 16S database, whereas ‘multi-affiliation’ refers to sequences found in the database, but with several possible taxa at the given taxonomic level (Fig. 4, Fig. 5B). The taxa considered relevant with their sparsity information were Proteobacteria (found in all participants), Oxalobacteraceae (found in 12/14 obese individuals and 8/15 lean individuals), and *Massilia* (found in 6/14 obese individuals and none of the lean individuals). The analysis revealed that the proportion of Proteobacteria was significantly higher in the livers of obese individuals than in those of lean individuals (81.0% [73.0–82.4%] *vs.* 74.3% [68.4–78.4%], *p* = 0.014) (Fig. 4C). We found no differences in the remaining phyla between the groups. At the family level, we found that the proportion of Oxalobacteraceae, which belong to Proteobacteria, was significantly higher in the obese group (up to 5% of the reads) than in the lean group (<0.007% of the reads) (Fig. 4C). This difference was reflected at the genus level, where *Massilia* from the family Oxalobacteraceae was found only in the obese group (up to 4.6% of the reads). For the remaining bacteria identified by the LEfSe algorithm, data were zero-inflated with differences between the groups driven by 1 or 2 outliers, and thus, they were disregarded (Fig. 4B).

### Similar bacterial metagenomic profiles in buffy coat from lean and obese individuals

The overall bacterial composition in buffy coat from the study group was mainly composed of Proteobacteria (82.8%) and Actinobacteria (10.4%) and, to a lesser extent, Acidobacteria (2.0%), Bacteroidetes (2.3%), and Firmicutes (2.5%) (Fig. 5A). The LEfSe algorithm identified no differences between the groups at either the phylum or order level. Differences at lower taxonomic levels were found to a limited extent (Fig. 5B) and were in all cases outlier driven (data not shown).

### The quantity of bacterial rDNA in the liver correlates with the FLI

**Fig. 6:**
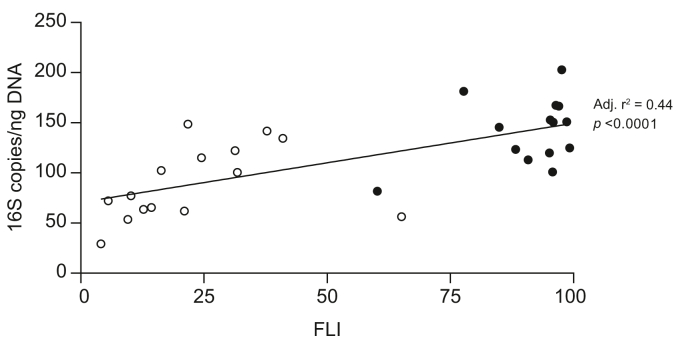
Association between the amount of bacterial 16S copies/ng DNA in the liver and the FLI. Scatter plot of the dependent variable (amount of bacterial 16S copies/ng DNA in liver samples) plotted against the significant independent variable (FLI) in lean (open symbols) and obese individuals (closed symbols). The trend line is shown together with the adjusted *r*^2^ value and the *p* value. FLI, fatty liver index.

Linear regression analyses were carried out and revealed correlations between the amount of bacterial 16S rDNA copies/ng DNA and BMI (adjusted *r*^2^ = 0.40, *p* <0.0001), waist–hip ratio (adjusted *r*^2^ = 0.32, *p* <0.0001), C-peptide (adjusted *r*^2^ = 0.42, *p* <0.0001), triglycerides (adjusted *r*^2^ = 0.18, *p* = 0.012), and FLI (adjusted *r*^2^ = 0.44, *p* <0.0001). Simple linear regressions were carried out between these parameters to avoid multicollinearity, and all of them correlated. Therefore, the final model was based only on FLI, as this parameter showed the strongest correlation with the amount of bacterial 16S rDNA copies/ng DNA in the liver samples (Fig. 6).

## Discussion

To our knowledge, this study presents the first characterisation of the liver microbiome in a cohort including healthy humans. The main findings include significantly higher amounts of bacterial rDNA, lower bacterial alpha diversity, and a higher proportion of Proteobacteria in liver biopsies from obese individuals compared with that from healthy lean controls. Additionally, we found a strong correlation between the amount of bacterial 16S rDNA copies/ng DNA in the liver and FLI, which is a validated marker of NAFLD. We propose that the increased bacterial load in the livers of obese individuals could constitute an early risk factor for the progression of NAFLD.

We found significantly higher amounts of bacterial 16S rDNA in liver biopsies from obese individuals compared with those from lean controls, both relative to the total amount of DNA and the weight of the samples (Fig. 1A,B). This implies that the difference between the groups was not a consequence of larger biopsy sample sizes or the number of cells acquired in the biopsies. It should be considered whether the difference could be a result of contamination during handling of the biopsies, from reagents, or introduction of bacteria during the biopsy procedure. However, the biopsies were collected using surgical procedures, which are put in place to avoid iatrogenic infections as a result of contamination, and each biopsy was handled with sterile equipment throughout. Additionally, contamination from reagents or the environment during the DNA extraction, amplification, and sequencing were minimised and controlled for in the pipeline, which is specifically optimised to analyse samples with a low abundance of bacterial DNA.^22^^,^^23^ A recent examination of the impact of environmental contamination on the applied pipeline from biopsy procedures and the handling of biopsies found that potential contamination during the sampling and handling of the samples was negligible relative to the specific signal measured in the samples, including liver biopsies.^24^

With an exploratory focus, we examined the liver microbiome in completely healthy lean individuals and in healthy individuals with obesity. The relatively small group sizes limit the statistical power of the study, and adjusting for multiple testing would likely reduce the number of significant findings, but not necessarily because they are false. This limitation, inherent to most exploratory studies, demands caution regarding the interpretation of our results. It should be noted that we did not culture liver tissue or blood, as it is not technically possible to culture most bacteria.^29^ Therefore, we are not able to discern whether the bacterial DNA in our samples reflects an indigenous living hepatic microbiome or represents remnants of degraded bacteria. Nevertheless, we found significant differences between lean and obese individuals, and, importantly, we show a higher proportion of DNA from lipopolysaccharide-carrying bacteria, which could lead to increased hepatic inflammation. Additionally, the study characterises the liver microbiome in hitherto unexamined populations and corroborates the findings from a recent study of the liver microbiome.^30^

NAFLD is considered the hepatic manifestation of the metabolic syndrome,^31^ and an increasing body of evidence suggests that increased translocation of bacteria and bacterial products from the gut to the liver via the portal circulation may contribute to NAFLD pathophysiology.^12^^,^^13^ This seems to be promoted by an energy-rich Western dietary pattern, which contributes to dysbiosis of the gut, entailing a change in the composition of bacteria in the intestinal lumen^8^^,^^9^ and disruption of the intestinal barrier integrity.^10^^,^^11^^,^^32^^,^^33^ Considering the position of the liver in relation to the gut and the portal circulation, our finding of significantly higher amounts of bacterial 16S rDNA in the livers of obese individuals supports the notion alluded to above. In the obese group, 13 of 14 individuals had hepatic steatosis, although unexpectedly only 7 met the criterion of NAFLD with ≥5% steatosis. We suggest that the increased bacterial load in the obese group could constitute an early risk factor in the development of NAFLD alongside factors of the metabolic syndrome (*i.e.* hepatic insulin resistance, hyperglycaemia, central obesity, hypertension, hypertriglyceridaemia, and low levels of HDL cholesterol^31^). In line with this, regression analyses revealed a strong correlation between the amount of bacterial 16S rDNA copies/ng DNA in the liver samples and FLI, which is a validated marker of NAFLD based on BMI, waist–hip ratio, gamma-glutamyltransferase, and triglycerides. However, the multifactorial aetiology of NAFLD is underlined by the microbial profiles of the 2 lean individuals with hepatic steatosis, which were similar to the rest of the lean group both quantitively and qualitatively (data not shown). Additionally, it should be stressed that we are not able to establish causality between the prevalence of hepatic steatosis and the quantity of bacterial rDNA in the obese group in this cross-sectional setting.

Metagenomic profiling of the liver biopsies revealed lower alpha diversity at the phylum level in the obese group compared with that in the lean group (Fig. 2A). Interestingly, lower species diversity of the gut microbiome (evaluated from faecal samples) has previously been reported in obesity,^34^^,^^35^ and our results could reflect a similar association. In addition to lower alpha diversity at the phylum level, we found a significantly higher proportion of Proteobacteria in the liver biopsies from obese individuals compared with those from lean controls (Fig. 4C). As mentioned, Proteobacteria are particularly interesting because of their membrane content of lipopolysaccharides, which are known to promote hepatic inflammation and fibrosis by activating Toll-like receptors on hepatic stellate cells and Kupffer cells.^13^^,^^36^ The position of the liver in relation to the portal venous system exposes it to the highest concentration of bacteria and/or bacterial products from the gut, and it has been proposed that the healthy liver acts as a filter that limits the concentration of pathogenic bacterial components in the systemic circulation.^20^^,^^37^ The quantitative and qualitative similarities of the bacterial rDNA in buffy coat between the groups in our study supports this and illustrate that the function seems maintained despite the presence of steatosis (Fig. 1, Fig. 5A,B). A longitudinal study previously identified the presence of Proteobacteria in blood as an independent risk marker for cardiovascular disease,^38^ thereby corroborating the association between this bacterial phylum and metabolic disease. Additionally, another study of the blood microbiome in patients with advanced stages of non-alcoholic fibrotic liver disease revealed significantly higher amounts of bacterial rDNA, lower alpha diversity, and an overabundance of Proteobacteria in this group, potentially as a result of impairment of normal liver function.^19^ Interestingly, we did not find any significant changes of the blood microbiome in our obese cohort with a high prevalence of hepatic steatosis (*i.e.* the earliest stage of NAFLD). Recently, Sookoian *et al.* reported an increase of Proteobacteria in the livers of patients with more advanced stages of liver disease.^30^ Despite the use of different techniques, our focus on early-stage NAFLD, and the inclusion of healthy individuals in the present study, our results and those of Sookoian *et al.* are corroboratory, supporting a Proteobacteria-enriched hepatic metataxonomic signature in NAFLD. Additionally, the results indicate that obesity-related changes in the liver microbiome are similar across different geographical regions. In the context of the abovementioned blood metataxonomic signatures in fibrotic liver disease, our results suggest that obesity-associated bacterial dysbiosis occurs in the liver at early stages of NAFLD and then may progress to the blood at more advanced stages of the disease, potentially as a result of impaired hepatic filtration of bacterial components.

In conclusion, we observed significantly higher amounts of bacterial rDNA in liver biopsies from obese individuals compared with those from healthy lean controls. Liver biopsies from the obese group were characterised by lower alpha diversity at the phylum level, and metagenomic profiling revealed a significantly higher proportion of Proteobacteria in these biopsies. To our knowledge, this is the first characterisation of the liver microbiome in healthy humans, and we speculate that the differences between lean and obese individuals could be a consequence of increased bacterial translocation from the gut to the liver, perhaps constituting an early risk factor for the development of more advanced liver disease alongside components of the metabolic syndrome.

## Financial support

This work was supported by The 10.13039/501100009708Novo Nordisk Foundation, The 10.13039/501100005747A.P. Møller Foundation, and Jacob and Olga Madsen’s Foundation. The funding sources did not have any influence on the study design; on the collection, analysis or interpretation of data; on the writing of the report; or on the decision to submit the article for publication.

## Authors’ contribution

Designed the study: MPS, JIB, AL, FKK*.* Collected the clinical data: MPS, MD, CS, MJK. Provided analyses: BL, AB. Drafted the manuscript: MPS. Reviewed/edited the manuscript: JIB, BL, AB, MD, MJK, CS, AL, TV, FKK*.* Conceived the study: FKK.

## Data availability statement

Data sets from the study are available from the corresponding author upon reasonable request.

## Conflicts of interest

The authors declare no conflicts of interest.

Please refer to the accompanying ICMJE disclosure forms for further details.

## References

[1] Younossi Z., Anstee Q.M., Marietti M., Hardy T., Henry L., Eslam M. (2018). Global burden of NAFLD and NASH: trends, predictions, risk factors and prevention. Nat Rev Gastroenterol Hepatol.

[2] Wong R.J., Aguilar M., Cheung R., Perumpail R.B., Harrison S.A., Younossi Z.M. (2015). Nonalcoholic steatohepatitis is the second leading etiology of liver disease among adults awaiting liver transplantation in the United States. Gastroenterology.

[3] Lindenmeyer C.C., McCullough A.J. (2018). The natural history of nonalcoholic fatty liver disease—an evolving view. Clin Liver Dis.

[4] Zoller H., Tilg H. (2016). Nonalcoholic fatty liver disease and hepatocellular carcinoma. Metabolism.

[5] Friedman S.L., Neuschwander-Tetri B.A., Rinella M., Sanyal A.J. (2018). Mechanisms of NAFLD development and therapeutic strategies. Nat Med.

[6] Kim D., Touros A., Kim W.R. (2018). Nonalcoholic fatty liver disease and metabolic syndrome. Clin Liver Dis.

[7] Abu-Shanab A., Quigley E.M.M. (2010). The role of the gut microbiota in nonalcoholic fatty liver disease. Nat Rev Gastroenterol Hepatol.

[8] Ley R.E., Turnbaugh P.J., Klein S., Gordon J.I. (2006). Microbial ecology: human gut microbes associated with obesity. Nature.

[9] Turnbaugh P.J., Ley R.E., Mahowald M.A., Magrini V., Mardis E.R., Gordon J.I. (2006). An obesity-associated gut microbiome with increased capacity for energy harvest. Nature.

[10] Brun P., Castagliuolo I., Leo V.D., Buda A., Pinzani M., Palù G. (2007). Increased intestinal permeability in obese mice: new evidence in the pathogenesis of nonalcoholic steatohepatitis. Am J Physiol Gastrointest Liver Physiol.

[11] Pendyala S., Walker J.M., Holt P.R. (2012). A high-fat diet is associated with endotoxemia that originates from the gut. Gastroenterology.

[12] Henao-Mejia J., Elinav E., Jin C., Hao L., Mehal W.Z., Strowig T. (2012). Inflammasome-mediated dysbiosis regulates progression of NAFLD and obesity. Nature.

[13] Seki E., Minicis S.D., Österreicher C.H., Kluwe J., Osawa Y., Brenner D.A. (2007). TLR4 enhances TGF-β signaling and hepatic fibrosis. Nat Med.

[14] Leung C., Rivera L., Furness J.B., Angus P.W. (2016). The role of the gut microbiota in NAFLD. Nat Rev Gastroenterol Hepatol.

[15] Tilg H., Cani P.D., Mayer E.A. (2016). Gut microbiome and liver diseases. Gut.

[16] Seki E., Schnabl B. (2012). Role of innate immunity and the microbiota in liver fibrosis: crosstalk between the liver and gut. J Physiol.

[17] De Minicis S., Rychlicki C., Agostinelli L., Saccomanno S., Candelaresi C., Trozzi L. (2014). Dysbiosis contributes to fibrogenesis in the course of chronic liver injury in mice. Hepatology.

[18] Païssé S., Valle C., Servant F., Courtney M., Burcelin R., Amar J. (2016). Comprehensive description of blood microbiome from healthy donors assessed by 16S targeted metagenomic sequencing. Transfusion (Paris).

[19] Lelouvier B., Servant F., Païssé S., Brunet A.-C., Benyahya S., Serino M. (2016). Changes in blood microbiota profiles associated with liver fibrosis in obese patients: a pilot analysis. Hepatology.

[20] Balmer M.L., Slack E., de Gottardi A., Lawson M.A.E., Hapfelmeier S., Miele L. (2014). The liver may act as a firewall mediating mutualism between the host and its gut commensal microbiota. Sci Transl Med.

[21] Schierwagen R., Alvarez-Silva C., Madsen M.S.A., Kolbe C.C., Meyer C., Thomas D. (2019). Circulating microbiome in blood of different circulatory compartments. Gut.

[22] Lluch J., Servant F., Païssé S., Valle C., Valière S., Kuchly C. (2015). The characterization of novel tissue microbiota using an optimized 16S metagenomic sequencing pipeline. PLoS One.

[23] Schierwagen R., Alvarez-Silva C., Servant F., Trebicka J., Lelouvier B., Arumugam M. (2020). Trust is good, control is better: technical considerations in blood microbiome analysis. Gut.

[24] Anhê F.F., Jensen B.A.H., Varin T.V., Servant F., Van Blerk S., Richard D. (2020). Type 2 diabetes influences bacterial tissue compartmentalisation in human obesity. Nat Metab.

[25] Suppli M.P., Bagger J.I., Lund A., Demant M., van Hall G., Strandberg C. (2020). Glucagon resistance at the level of amino acid turnover in obese subjects with hepatic steatosis. Diabetes.

[26] Suppli M.P., Rigbolt K.T.G., Veidal S.S., Heebøll S., Eriksen P.L., Demant M. (2019). Hepatic transcriptome signatures in patients with varying degrees of nonalcoholic fatty liver disease compared with healthy normal-weight individuals. Am J Physiol Gastrointest Liver Physiol.

[27] Eriksen P.L., Vilstrup H., Rigbolt K., Suppli M.P., Sørensen M., Heebøll S. (2019). Non-alcoholic fatty liver disease alters expression of genes governing hepatic nitrogen conversion. Liver Int.

[28] Escudié F., Auer L., Bernard M., Mariadassou M., Cauquil L., Vidal K. (2018). FROGS: find, rapidly, OTUs with galaxy solution. Bioinformatics.

[29] Stewart E.J. (2012). Growing unculturable bacteria. J Bacteriol.

[30] Sookoian S., Salatino A., Castaño G.O., Landa M.S., Fijalkowky C., Garaycoechea M. (2020). Intrahepatic bacterial metataxonomic signature in non-alcoholic fatty liver disease. Gut.

[31] Marchesini G., Bugianesi E., Forlani G., Cerrelli F., Lenzi M., Manini R. (2003). Nonalcoholic fatty liver, steatohepatitis, and the metabolic syndrome. Hepatology.

[32] Miele L., Valenza V., Torre G.L., Montalto M., Cammarota G., Ricci R. (2009). Increased intestinal permeability and tight junction alterations in nonalcoholic fatty liver disease. Hepatology.

[33] Volynets V., Küper M.A., Strahl S., Maier I.B., Spruss A., Wagnerberger S. (2012). Nutrition, intestinal permeability, and blood ethanol levels are altered in patients with nonalcoholic fatty liver disease (NAFLD). Dig Dis Sci.

[34] Turnbaugh P.J., Hamady M., Yatsunenko T., Cantarel B.L., Duncan A., Ley R.E. (2009). A core gut microbiome in obese and lean twins. Nature.

[35] Le Chatelier E., Nielsen T., Qin J., Prifti E., Hildebrand F., Falony G. (2013). Richness of human gut microbiome correlates with metabolic markers. Nature.

[36] Rivera C.A., Adegboyega P., van Rooijen N., Tagalicud A., Allman M., Wallace M. (2007). Toll-like receptor-4 signaling and Kupffer cells play pivotal roles in the pathogenesis of non-alcoholic steatohepatitis. J Hepatol.

[37] Bellot P., García-Pagán J.C., Francés R., Abraldes J.G., Navasa M., Pérez-Mateo M. (2010). Bacterial DNA translocation is associated with systemic circulatory abnormalities and intrahepatic endothelial dysfunction in patients with cirrhosis. Hepatology.

[38] Amar J., Lange C., Payros G., Garret C., Chabo C., Lantieri O. (2013 Jan 25). Blood microbiota dysbiosis is associated with the onset of cardiovascular events in a large general population: the D.E.S.I.R. study. PloS One.

