# Supplementary material for: Hepatic microbiome in healthy lean and obese humans
Source: JHEP Rep. 2021 Apr 27;3(4):100299. doi: 10.1016/j.jhepr.2021.100299 (PMC8207208; doi:10.1016/j.jhepr.2021.100299)
Supplement: Multimedia component 1 [file mmc1.pdf]

Hepatic microbiome in healthy lean and obese humans

Malte Palm Suppli, Jonatan Ising Bagger, Benjamin Lelouvier, Amandine Broha, Mia Demant, Merete Juhl Kønig, Charlotte Strandberg, Asger Lund, Tina Vilsbøll, Filip Krag Knop

Table of contents

Fig. S1..2

Fig. S2..3

Fig. S3..4

Fig. S4..5

SUPPLEMENTARY FIGURES

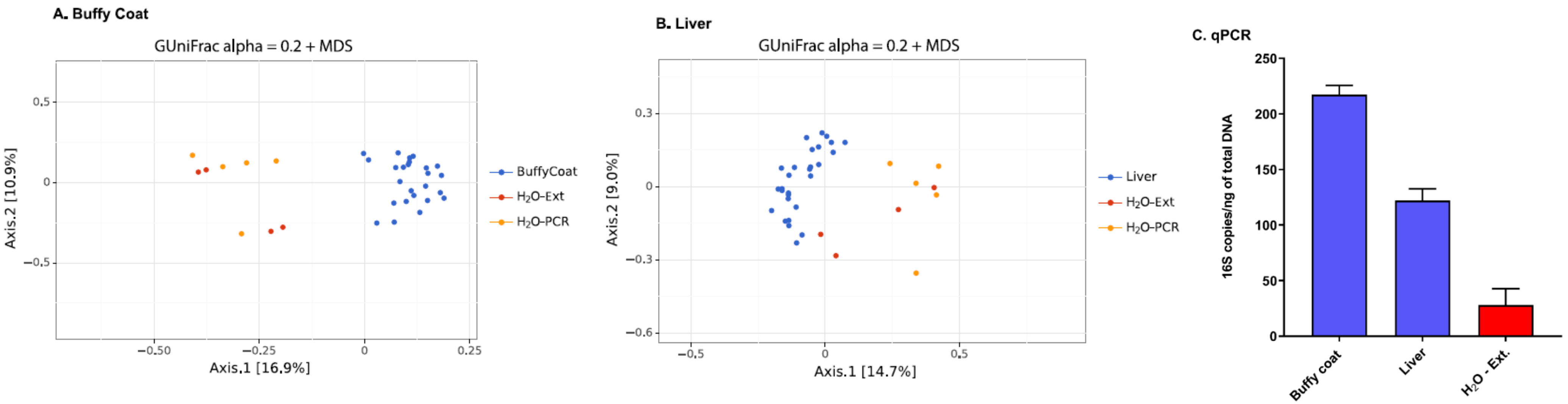

Fig. S1. A: Comparison of beta diversities by ordination analysis (based on Generalised UniFrac (GUniFrac) distances) of the 16S sequencing of the buffy coat samples and negative controls (H_2O-PCR and H_2O-Ext). B: Comparison of beta diversities by ordination analysis (based on GUniFrac distance) of the 16S sequencing of the liver samples and negative controls (H_2O-PCR and H_2O-Ext). C: Comparison of the quantity of bacterial DNA assessed by qPCR in the buffy coat samples, liver samples and negative controls (H_2O-Ext). H_2O-Ext: molecular grade water added in an empty tube, extracted, amplified and sequenced at the same time as the samples; H_2O-PCR: molecular grade water added in an empty tube and amplified and sequenced at the same time as the extracted DNA of the samples; MDS: multidimensional scaling

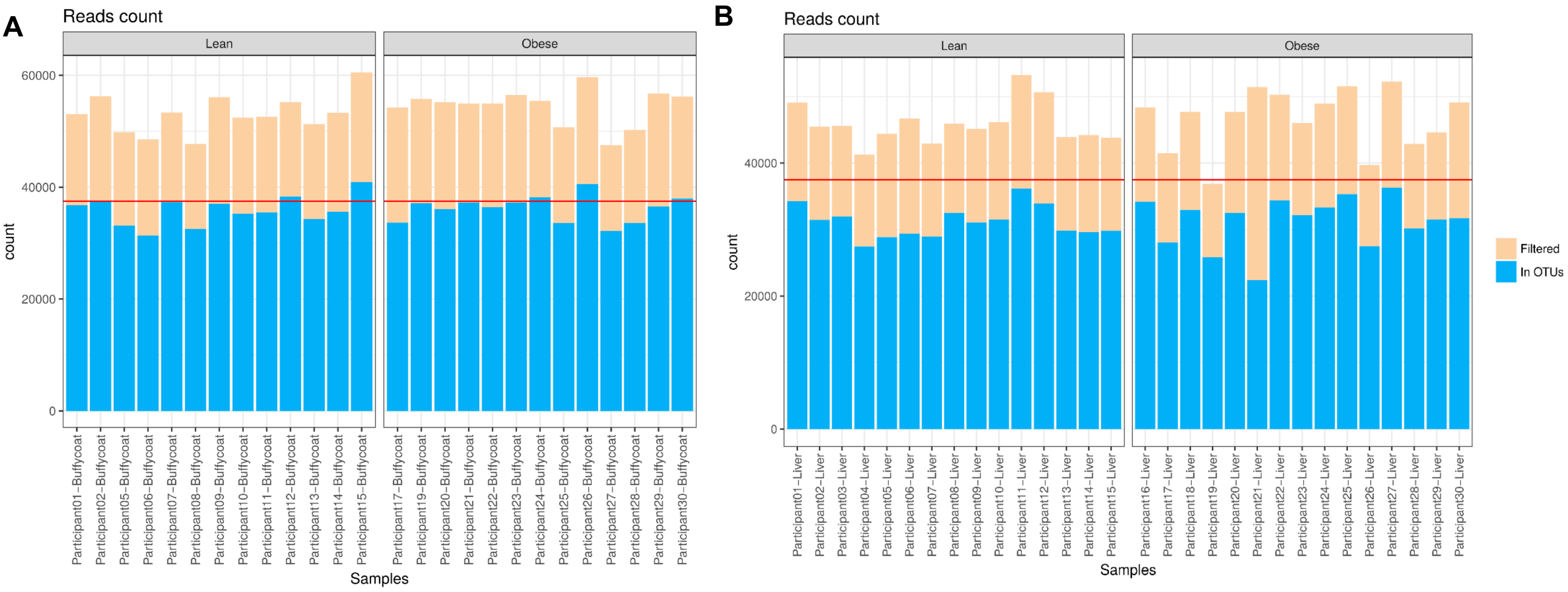

Fig. S2. The total number of sequences (merged pair reads) per sample (blue + orange), filtered from the data (orange) and clustered in operational taxonomic units (OTUs) (blue) in the liver samples (A) and in the buffy coat samples (B). The red line corresponds to the optimal number of reads (orange + blue) targeted by the pipeline.

A

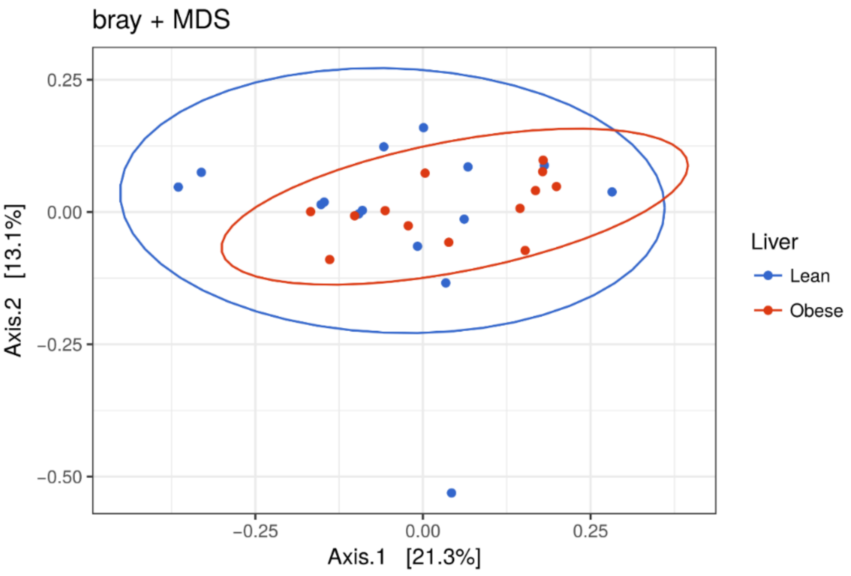

B

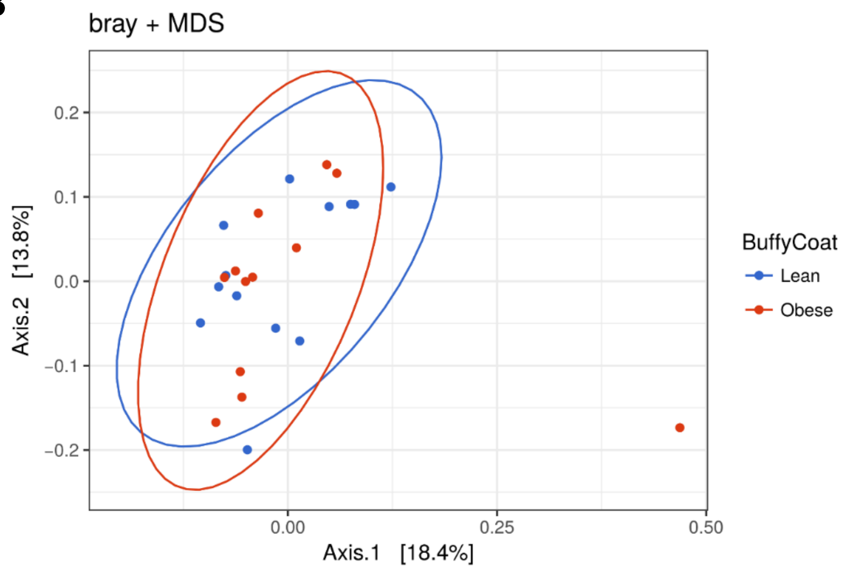

Fig. S3. Principal component analysis (ordination) of the 16S rRNA gene sequencing data using the Bray-Curtis dissimilarity measure in the liver samples (A) and in the buffy coat samples (B). MDS: multidimensional scaling.

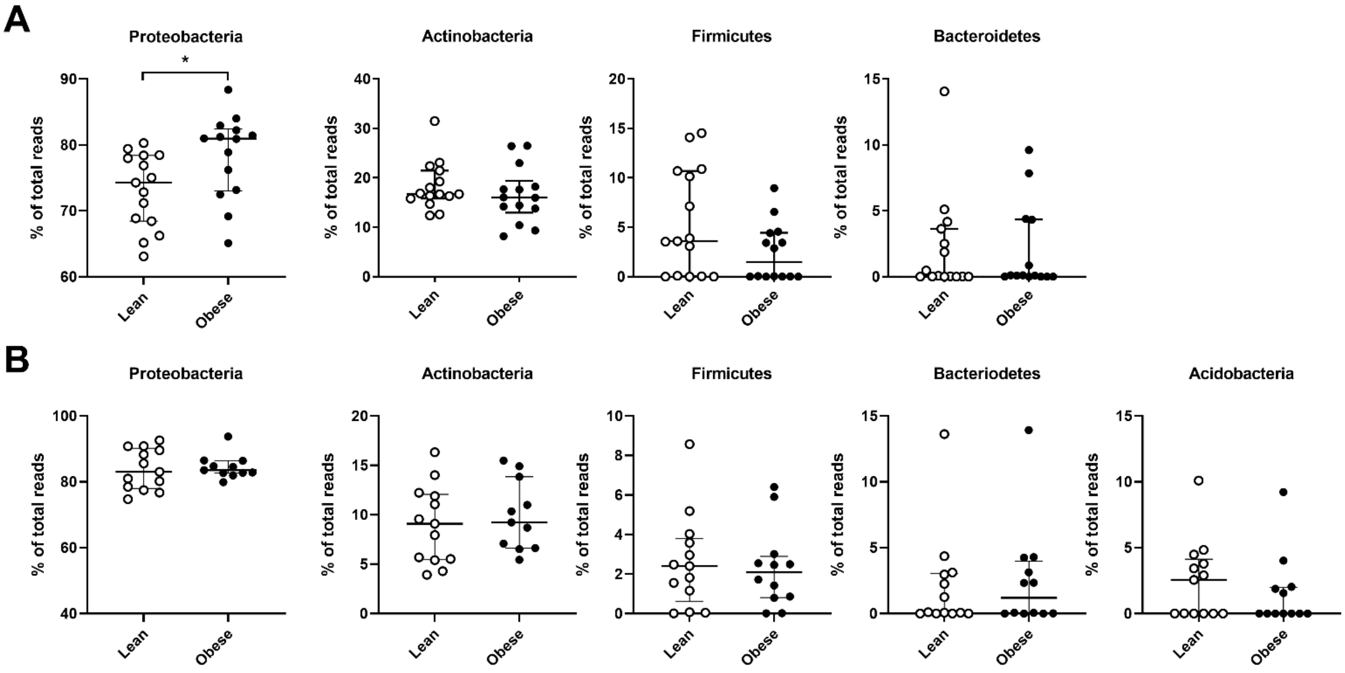

Fig. S4. Median ± interquartile range (whiskers plot) and individual values (dot plot) of bacterial profiles at phylum level assessed by 16S metagenomics sequencing in the liver samples (A) and in the buffy coat samples (B) from lean individuals (open symbols) and obese individuals (filled symbols). * $P < 0.05$ (Mann-Whitney U test).
