# Supplementary material for: Hepatic microbiome in healthy lean and obese humans
Source: JHEP Rep. 2021 Apr 27;3(4):100299. doi: 10.1016/j.jhepr.2021.100299 (PMC8207208; doi:10.1016/j.jhepr.2021.100299)
Supplement: Multimedia component 2 [file mmc2.pdf]

JHEP|Reports

JHEP Reports

CTAT methods

Tables for a "Complete, Transparent, Accurate and Timely account" (CTAT) are now mandatory for all revised submissions. The aim is to enhance the reproducibility of methods.

- Only include the parts relevant to your study
- Refer to the CTAT in the main text as 'Supplementary CTAT Table'
- Do not add subheadings
- Add as many rows as needed to include all information
- Only include one item per row

If the CTAT form is not relevant to your study, please outline the reasons why:

1.1 Antibodies

Name	Citation	Supplier	Cat no.	Clone no.

1.2 Cell lines

Name	Citation	Supplier	Cat no.	Passage no.	Authentication test method

1.3 Organisms

Name	Citation	Supplier	Strain	Sex	Age	Overall n number

1.4 Sequence based reagents

Name	Sequence	Supplier
16S Primer forward	5'-TCCTACGGGAGGCAGCAGT-3'	N/A
16S Primer reverse	5'-GGACTACCAGGGTATCTAATCCTGTT-3'	N/A

1.5 Biological samples

Description	Source	Identifier
Liver biopsy specimens	Human	

1.6 Deposited data

Name of repository	Identifier	Link

1.7 Software

Software name	Manufacturer	Version
R	R software	3.5.2
GraphPad Prism	GraphPad Software	8.0.0
FROGS	N/A	1.40 beta
vsearch	N/A	1.9.5
swarm	N/A	2.1.6
Blast+	N/A	2.2.30
PhyloSeq	N/A	1.14.0
Databank Silva	Max Planck Institute for Marine Microbiology and Jacobs University, Bremen, Germany	128 Parc

1.8 Other (e.g. drugs, proteins, vectors etc.)

1.9 Please provide the details of the corresponding methods author for the manuscript:

Malte Palm Suppli
Gentofte Hospital
Gentofte Hospitalsvej 7, 3rd floor
2900 Hellerup, Denmark

e-mail: malte.palm.suppli@regionh.dk
phone: +45 25151436

2.0 Please confirm for randomised controlled trials all versions of the clinical protocol are included in the submission. These will be published online as supplementary information.

